# Mesolithic projectile variability along the southern North Sea basin (NW Europe): Hunter-gatherer responses to repeated climate change at the beginning of the Holocene

**DOI:** 10.1371/journal.pone.0219094

**Published:** 2019-07-17

**Authors:** Philippe Crombé

**Affiliations:** Archaeology Department, Ghent University, Ghent, Belgium; Max Planck Institute for the Science of Human History, GERMANY

## Abstract

This paper investigates how former hunter-gatherers living along the southern North Sea coast in NW Europe adapted to long-term and short-term climatic and environmental changes at the beginning of the Holocene. It is argued that contemporaneous hunter-gatherers repeatedly changed their hunting equipment in response to changing climate and environment, not just for functional reasons but mainly driven by socio-territorial considerations. Based on a Bayesian analysis of 122 critically selected radiocarbon dates a broad chronological correlation is demonstrated between rapid changes in the design and technology of stone projectiles and short but abrupt cooling events, occurring at 10.3, 9.3 and 8.2 ka cal BP. Combined with the rapid sea level rises and increased wildfires these climatic events probably impacted the lifeways of hunter-gatherers in such a way that they increasingly faced resource stress and competition, forcing them to invest in the symbolic defense of their social territories.

## Introduction

**Fig 1:**
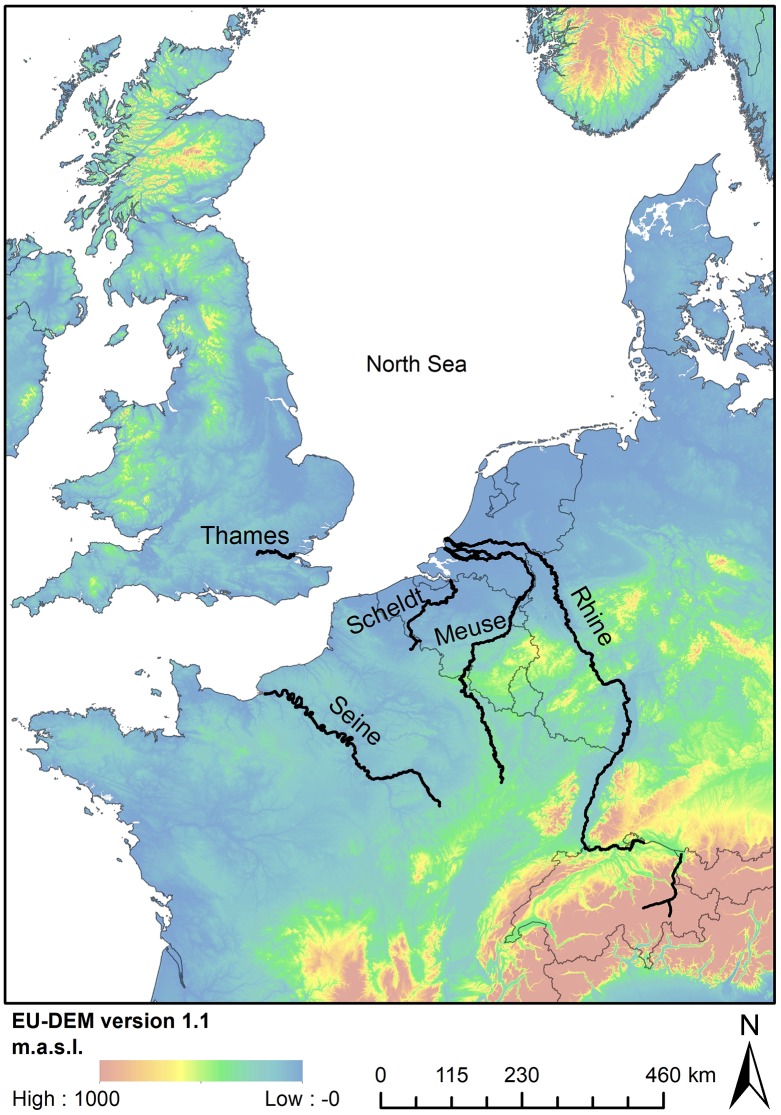
Elevation map (European Union, Copernicus Land Monitoring Service 2019, European Environment Agency (EEA)) with indication of the main rivers in the Rhine-Meuse-Scheldt region.

The Early Holocene in Europe archaeologically corresponds to the Middle Stone Age or the Mesolithic (maximum duration ca. 11,000–6000 cal BP). Due to rapid climatic amelioration, contemporaneous hunter-gatherers faced similar environmental changes as we encounter today, among which a significant rise of the sea level [1], a northwards migration of particular plant and animal species [2], the concomitant extinction of less thermophilous species, and increased drought [3–4] and wildfires [5]. For many years, archaeologists have been studying how these ecosystem changes impacted the lifeways of Mesolithic hunter-gatherers. Since in most European countries organic remains, such as animal bones, seeds and fruits, are only rarely preserved in Mesolithic sites, due to soil acidity, this research has mainly focused on the lithic industries. One specific stone tool, the microlith named after its small sizes (mean length 2-3cm), has been studied intensively. Based on microscopic use wear analyses and experimental research [6–9] microliths are generally considered part of the hunting gear, more specifically as stone arrowheads and barbs originally mounted on wooden arrowshafts. During the Mesolithic a wide variety of microlithic forms, ranging from purely geometric armatures (triangles, crescents, trapezes) to simple backed points have been developed and used. Long-term inter- and intrasite comparative research on a regional [10–11] and European scale [12–13] has identified several taxonomic groups or assemblage-types (AT), each characterized by one or two dominant microlithic types. The interpretation of this typological variability has long been hampered by dating problems, related to the use of poorly associated samples and/or samples with a potentially large inbuilt age (e.g. charcoal) for radiocarbon dating. Thanks to recent improvements in sample selection and the technique of radiocarbon dating (AMS, refined preparation techniques, etc.), during the last few decades relatively detailed typo-chronologies could be established for most European regions, demonstrating in most cases that the different ATs are regional diachronic adaptations and refinements of the hunting equipment and strategies in response to changing environments [14–17]. One of these regions is the Rhine-Meuse-Scheldt (RMS) area along the southern North Sea basin in NW Europe, encompassing northern France (north of the Seine), Belgium, the southern Netherlands (south of the Meuse/Rhine), and western Germany (east of the Rhine) (Fig 1). Intensive research of the last two decades has provided a considerable amount of radiocarbon dates, allowing the study of the correlation between microlith evolution and environmental changes in greater detail.

## Materials and methods

A database of all radiocarbon dates available from Mesolithic sites in the RMS region has been compiled. The database also includes some radiocarbon dates from southern UK, specifically those related to ATs which present some affinities with those of the RMS region (cf. Table 1). In view of limiting the effects of old wood and contamination the present study only uses the most reliable dates from this list (n = 228), including dates made on carbonized short-lived plant material (mainly charred hazelnut shells), charcoal from clear anthropogenic features (e.g. hearths, storage-pits, etc.), unburnt bone and organic residue on flint artefacts (e.g. resin) [18]. Despite their large number, charcoal dates performed on scattered fragments or samples from natural and/or undetermined features (e.g. “hearth-pits”, wind-throw pits, etc.) are considered unreliable mainly due to their insecure association [18–19]. For the time being, dates on calcined human and animal bones, although often found in close anthropogenic association, resp. burials [20] and domestic surface-hearths [21], are not incorporated given their insufficient age control, as a result of e.g. the possible carbon exchange from the firewood during the burning process and/or taphonomic factors [22–23].

**Table 1:** Overview and correspondence of the main ATs identified within the different research areas of the Rhine-Meuse-Scheldt region.

	Early Mesolithic				Middle Mesolithic		Late Mesolithic	
Belgium/S. Netherlands/W Germany [24–25]	Neerharen	Ourlaine	Verrebroek	Chinru	Sonnisse Heide	Gelderhorsten	Paardsdrank	Ruiterskuil
N. France [26]	Early Maglemose	Beuronian with crescents	Beuronian with scalene triangles		RMS-A		RMS-B	
Southern UK [14]	Deepcar	-	-	-	Honey Hill?		-	-
Dominant microlith type	Point with unretouched base	Crescent	Scalene triangle (+ pt. unret. base)	Scalene triangle (+ pt. ret. base)	Small backed bladelets (+ invasively retouched microlith)	Invasively retouched microlith (+ small backed bladelet)	Trapeze (45–65%)	Trapeze (>75%)

**Fig 2:**
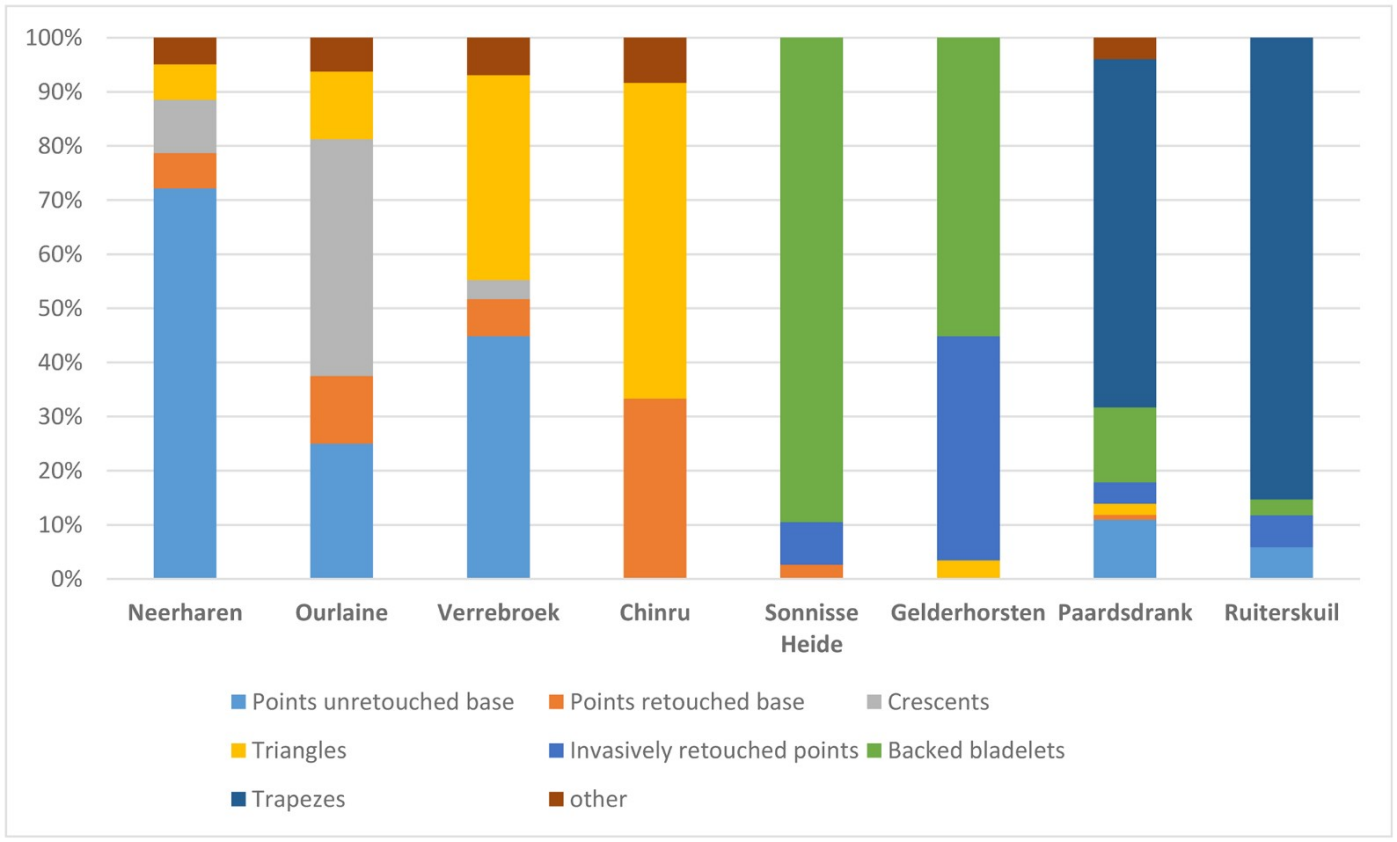
Microlith composition of the eight ATs defined within the Rhine-Meuse-Scheldt region.

The selected dates were subsequently allocated to one of the Mesolithic ATs, identified in earlier studies for the RMS area (Table 1; Fig 2). Therefore, more than half of the dates (n = 126) could be securely associated (S1 Table), the majority with ATs belonging to the Early Mesolithic. The fewer radiocarbon dates for the Middle and Late Mesolithic ATs reflect the limited number of excavated sites and the reduced occurrence of reliable dating samples, in particular charred hazelnut shells.

Based on this set of dates, a statistical analysis using Bayesian modelling [27], available from the online OxCal program version v4.3, was performed. All dates were calibrated according to the IntCal13 atmospheric calibration curve [28]. Bayesian modelling was done using the “Overlapping Phase” function, in order to calculate the start and end of each AT. The Agreement Index was used for selecting the most reliable dates; dates with an Agreement Index (AI) below 60% were considered as outliers and eliminated from the model [29].

## Results

**Fig 3:**
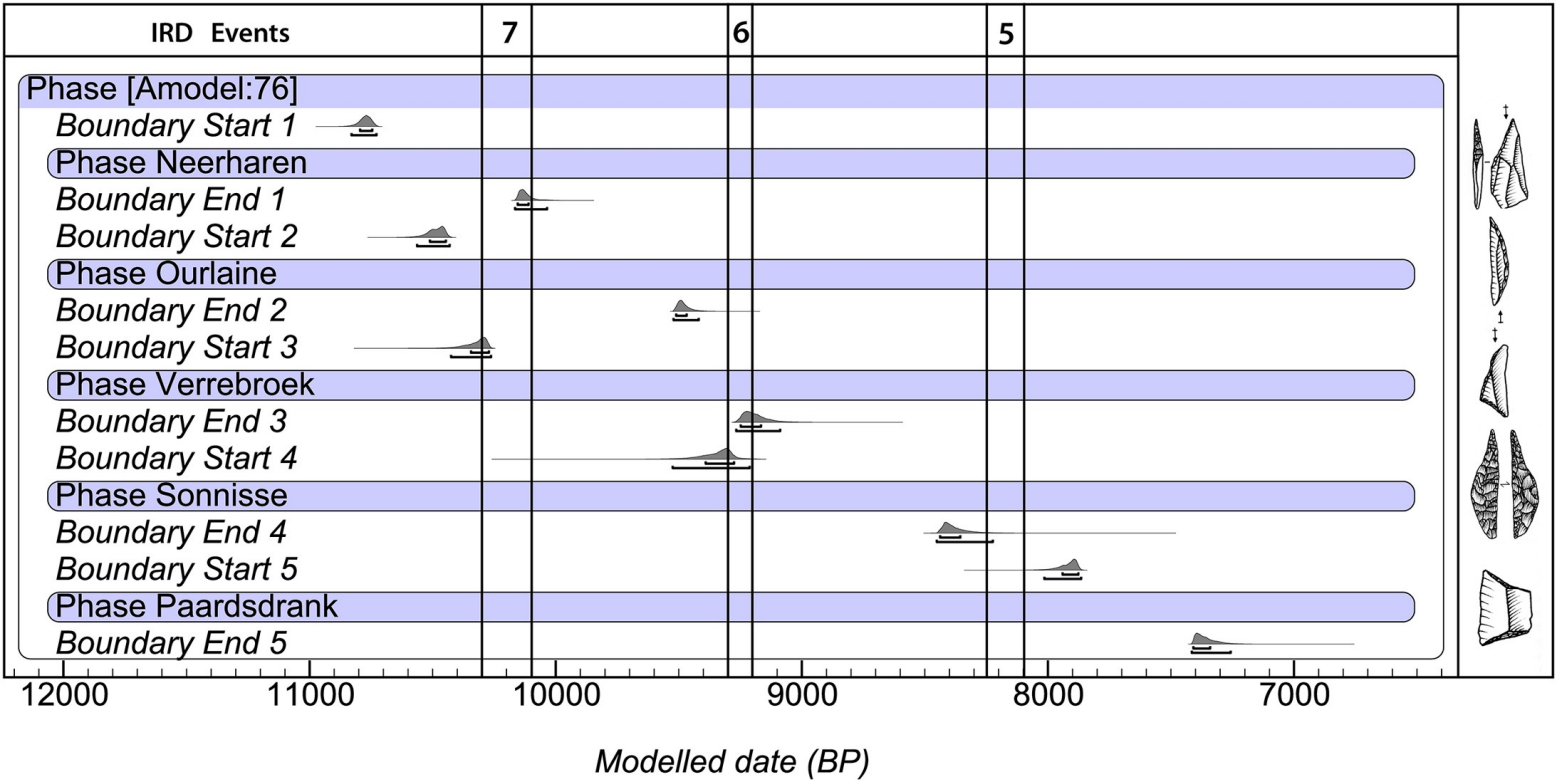
Modelled start and end date of the different ATs and their associated microlithic forms. The chronology of the ERD-events is based on [30–31].

A first model (S2 Table) reached a poor overall agreement (A_overall_ = 44.4%), mainly due to the presence of two outliers (AI<10%). Elimination of these resulted in a second model (S3 Table) with an acceptable A_overall_ (70.3%), however still including two dates which just do not reach the level of acceptance (AI = 47/50%). Without the latter, a final model with an A_overall_ of 80.6% and no outliers was obtained (S4 Table; Fig 3).

Contrary to the prevailing theories, the model shows the existence of a considerable chronological overlap between different ATs, proving that these do not merely reflect diachronic adaptions (Table 2). This is particularly the case for the Early Mesolithic “Neerharen”, “Ourlaine” and “Verrebroek/Chinru” ATs. Although there is clearly a difference in their starting date, all three coexisted during the last quarter of the 11th millennium cal BP. The chronological overlap between the “Ourlaine”, dominated by crescents, and the “Verrebroek/Chinru” AT with mainly scalene triangles, is considerable as it spans almost an entire millennium, from the mid-11th until the mid-10th millennium cal BP. In the course of the 2nd half of the 10^th^ millennium both were fully replaced by the “Sonnisse Heide” and “Gelderhorsten” ATs, characterized by new microlithic types, i.e. invasively retouched microliths and small backed bladelets. The former differed radically from previous geometric microliths both on a morphological level, with the introduction of new mistletoe and leaf-shaped forms, as well as on a technical level by the use of flat, locally (e.g. at the base and/or tip) bifacial retouches instead of direct, steep retouches. A similar abrupt change in microlith form and technology occurred in the last quarter of the 9^th^ millennium cal BP and the transition towards the 8^th^ millennium cal BP, with the appearance of the first trapezes, formed by a double truncation of regular blade(let)s. Although our model indicates a start date between ca. 8015–7865 cal BP (95% probability) and probably 7940–7875 cal BP (65% probability), based on the second model (S3 Table) a slightly earlier appearance (8300–8050 or 8215–8065 cal BP) cannot be excluded.

**Table 2:** Results of the Bayesian “Phase” modelling (model 3; A_overall_ 80.6%).

AT (N of dates)	start	end	start	end
	68.2% probability		95.4% probability	
Neerharen (32)	10795–10746	10155–10110	10830–10729	10165–10035
Ourlaine (34)	10511–10447	9511–9468	10563–10431	9521–9419
Verrebroek/Chinru (27)	10344–10272	9248–9166	10425–10263	9267–9088
Sonnisse Heide/Gelderhorsten (15)	9391–9276	8438–8356	9525–9212	8452–8224
Paardsdrank/Ruiterskuil (14)	7941–7876	7409–7341	8015–7865	7415–7258

## Discussion

**Fig 4:**
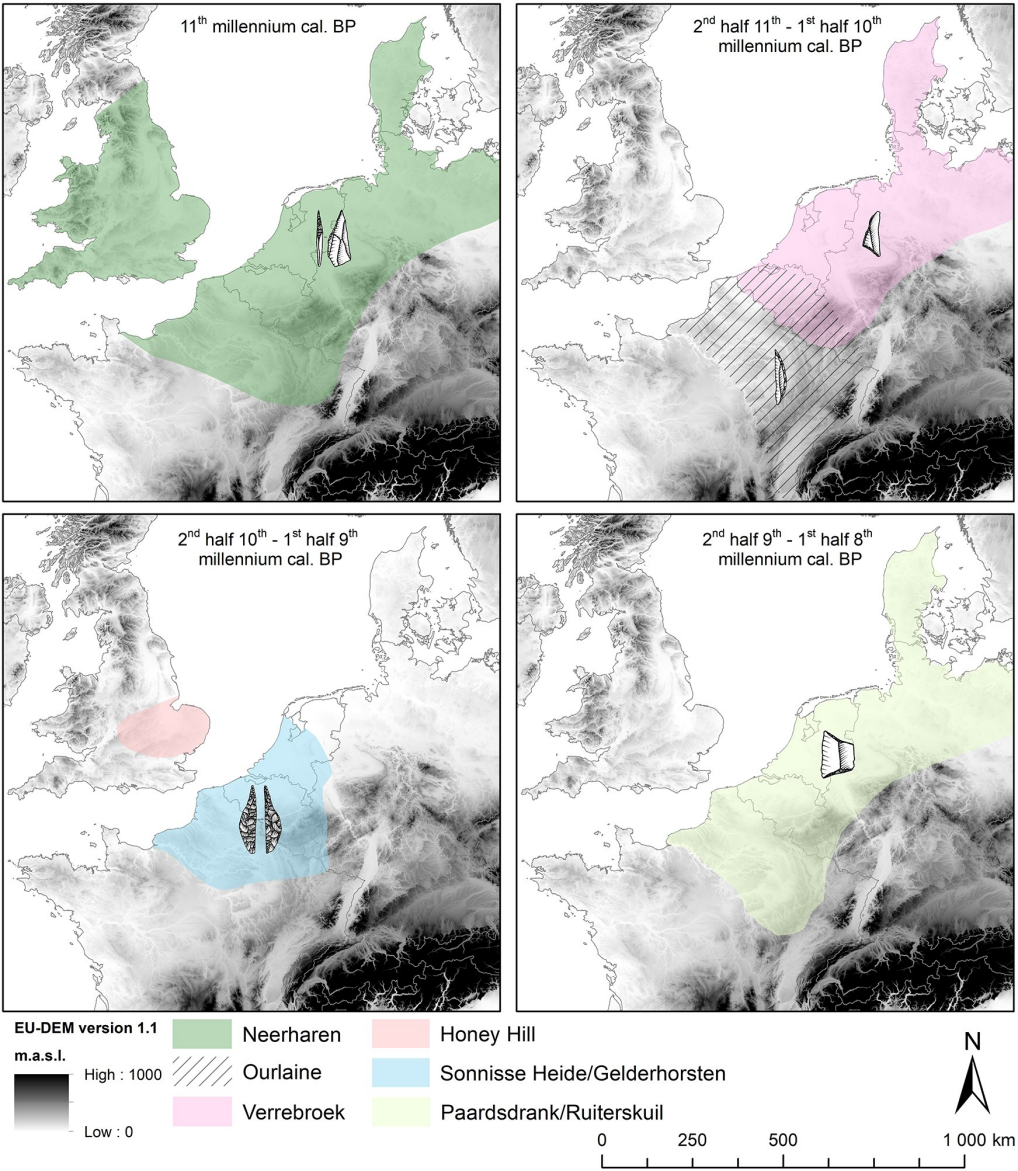
General distribution maps of the ATs within the RMS region. Source map (European Union, Copernicus Land Monitoring Service 2019, European Environment Agency (EEA)).

The high-resolution radiocarbon evidence from the RMS area demonstrates that the significance of the microlithic variability within the European Mesolithic is much more complex than previously acknowledged [15, 26, 32–33]. The chronological model clearly proves the co-existence of at least two different sets of projectiles during the late 11th to mid-10th millennium cal BP. Use-wear analysis of the dominant microlith types within the “Ourlaine” and “Verrebroek/Chinru” ATs, crescents and scalene triangles respectively, have pointed out their almost exclusive use as stone barbs [7, 8, 34]. These could have been combined with a stone tip, most likely a backed point or a point with retouched base, or hafted on a wooden arrow shaft sharpened at one end (slotted arrows). The scarcity of faunal remains, in particular for the “Verrebroek/Chinru” AT, hinders the assessment of whether these different arrow compositions express variations in hunting practices, such as differences in hunted game species and/or size (e.g. large versus small game) or in hunting season (e.g. dry versus wet season hunting), as observed in ethnographical contexts [35, 36]. Alternatively, the two arrow type compositions may also be an expression of social differences, each set representing a specific social or ethnic group. The use of projectiles, along with other objects such as ornaments [37, 38], as a means to visualize group affiliation, named “emblematic style” (versus “assertive style”), is well-documented among (sub)recent hunter-gatherers [39, 40]. On a larger scale both ATs are part of two geographically distinct techno-complexes [12, 13] (Fig 4): the “Verrebroek/Chinru” AT has strong affinities with the Maglemosian/Duvensian techno-complex, covering almost the entire North-European Plain from the North Sea coast up to Poland and southern Scandinavia, while the “Ourlaine” AT has clearly a more southern connection with the Beuronian techno-complex, more specifically with the northern Beuronian, characterized by numerous crescents [15]. The latter is bound to the Paris Basin and eastern France [41]. It is very tempting to interpret this geographical patterning in terms of two distinct population groups, e.g. on the level of language-families, though this remains difficult to prove. Interestingly, the RMS region is situated at the contact between both techno-complexes, explaining the co-occurrence of both ATs. This is particularly so for the Belgian territory, where both ATs occur within the same river basins (Scheldt and Meuse) and even within the same site (e.g. Verrebroek, S1 Table). On the other hand, co-occurrence is less prominent in the southern Netherlands and northern France; the Ourlaine AT is hardly known in the former, while in the Paris basin triangle-dominated assemblages are very rare and date to the final stage of the “Verrebroek/Chinru” ATs (e.g. Saleux, S1 Table) [42]. Following the hypothesis of two distinct ethnic groups, this would imply an overlap of their territories corresponding roughly to present Belgium (Fig 4). Interestingly, precisely in this overlapping region an exchange of specific exotic raw materials is attested. Two quartzite types, Wommersom and Tienen quartzite, are used for the production of projectile implements, however the former was restricted for the production of crescents while the latter was mainly used for triangle production [43, 44]. As both quartzites originate from the same outcrop area in the middle of Belgium, this pattern clearly reflects a deliberate choice of two different social groups within the same territory, who possibly shared overlapping annual ranges.

**Fig 5:**
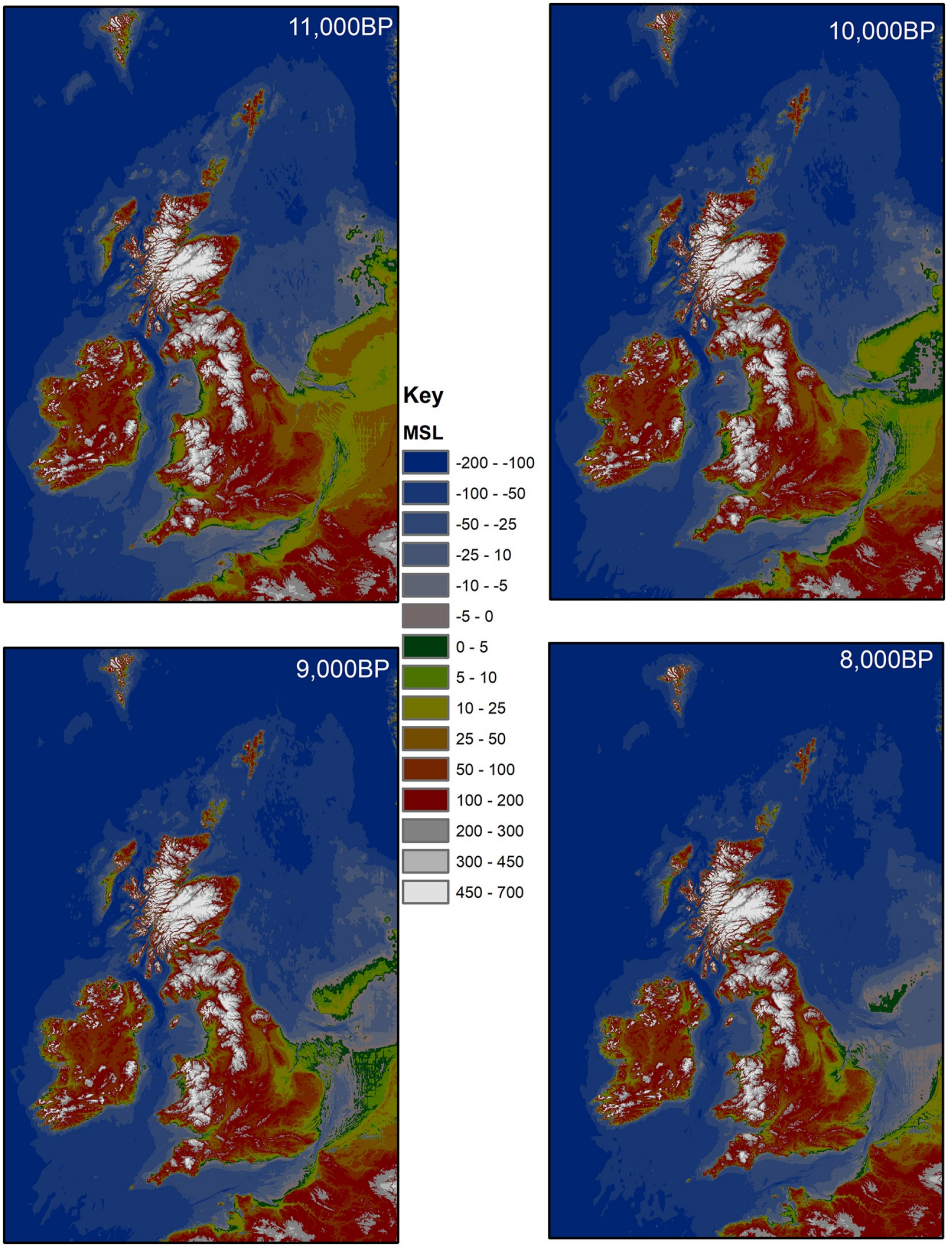
Palaeogeographic models of the North Sea drowning from 11,000 to 8000 cal BP. Map produced in part from Ordnance Survey Digimap, SeaZone solutions and GEBCO 08 (www.gebco.net) data. Produced by F. Sturt (University of Southampton).

Considering that projectile style and raw material may have been used during the late 11th to mid-10th millennium cal BP for communicating social group membership, it is important to investigate the context in which this occurred. Ethnographically, the symbolization of group membership has often been connected with increased territoriality, pointing to the exclusive use of resources or exploitation of an area [45–47], and preventing neighboring groups’ members from gaining access to these resources without permission. These situations mostly emerge in environments with abundant and dense food resources that are predictable in time and space and are worth defending, such as coastal areas. This was most likely not the case during the Early Holocene in the RMS area. According to various palynological studies [48–50] the Early Holocene climatic amelioration triggered a rapid colonization of trees, initially birch (Preboreal) and later on pine and hazel (first half of Boreal), forming rather open forests with stable and evenly spaced, but less predictable animal (e.g. wild boar, red and roe deer, auroch) and plant resources. Except perhaps for edible marshy plants such as bulrush (*Typha latifolia*), club-rushes (*Schoenoplectus* spp.), and common reed (*Phragmites australis*) [51], which may have clustered along former river banks, environmental conditions were far from comparable to the ethnographic contexts characterized by a high degree of territoriality. Yet, ethnography also shows that territoriality occasionally occurs in forested environments with scattered resources, in particular in situations of resource competition and stress. The latter can be caused by a temporal increase of the population density and/or a reduction of the carrying capacity of the environment [45, 47]. This scenario might be applicable to the RMS area in the Early Holocene, as the area was facing one of its most dramatic environmental changes related to rapid sea level rises (Fig 5). According to recent modelling [52] ca. 50.000 km^2^ of the North Sea basin was drowned between 11,000 and 9500 cal BP, which ethnographically corresponds to the territory of an entire dialect tribe living in a forested environment with stable, evenly spaced resources, generally comprising 300–500 members. The former habitants of the North Sea land-bridge may thus have been forced to gradually leave their territories by moving to the south(east) and (north)west in direction of land already occupied by other groups. This is supported by recent stable isotope analyses on Mesolithic skeleton remains from the North Sea bed [53], which clearly demonstrate that the last Doggerland inhabitants hardly exploited marine resources and hence did not adapt their diet to the new coastal/marine environment. Population movement might also explain the co-occurrence of the “Ourlaine” and “Verrebroek/Chinru” ATs in the RMS region, the former representing the original population while the latter reflects the incoming hunter-gatherers from the drowned North sea basin. It could also explain the rather late introduction of triangles into the Paris basin, situated at the southern limit of the RMS. In addition, a comparable contemporaneity between the Early Holocene ATs, named the “Starr Car”,” Deepcar” and “Horsham” ATs, during the 2^nd^ half of the 11^th^ and 1^st^ half of the 10^th^ millennium cal BP has recently also been identified in southern England [54], indicating that probably both sides of the channel faced similar challenges as a result of population displacements. This idea of population increase during the Early Holocene along the southern North Sea basin at first sight seems to be supported by the sharp increase and absolute predominance of radiocarbon dates and sites attributed to the Early Mesolithic in comparison to the Middle and Late Mesolithic. Applying the principles of “dates as data” to the ^14^C-dataset, a summed probability distribution curve emerges presenting an important peak of radiocarbon dates in the 11^th^ and 1^st^ half of the 10^th^ millennium cal BP [55]. Similarly in many regions of the RMS a substantial increase in sites is observed at the start of the Mesolithic [44]. However, these trends in radiocarbon dates and sites might be biased to a certain degree, as a result of taphonomic processes, research foci and sampling among others (cf. Materials and Methods; 55). In addition, these trends might also be caused by other factors, such as changes in settlement system (forager versus collector system) and residential mobility in response to changing environments at the start of the Mesolithic [56]. Finally, it should be mentioned that peaks in ^14^C dates and sites during the Early Mesolithic have also been reported in more inland regions of Europe, such as southern Germany [57], indicating that the interpretation of these data is not straightforward and reconstructions of palaeodemography challenging.

However, sea level rises may not have been the only trigger for increased boundary defense during the late 11th to mid-10th millennium. The radiocarbon evidence (Fig 3) indicates that the massive introduction of triangles at the start of the “Verrebroek/Chinru” AT occurred around the timing of a short but abrupt cooling event known as the 10.3 ka event [30], also known as the IRD (Ice Rafted Debris) 7 event [58] or Erdalen event [59], dated between ca. 10,300 and 10,100 cal BP. Although strict synchronicity between both cannot be demonstrated due to the chronological variability of the different palaeoclimate proxies and the relative chronological resolution of the ATs, a causality is a real possibility. The precise impact of this punctuated climatic event on the ecosystem in the southern North Sea area remains unclear due to a lack of high-resolution palaeoecological analyses [44], yet in western Scandinavia it corresponded to a marked increase of winter precipitation (mainly snow) and a major advance of glaciers [60]. The only detailed palaeoenvironmental records so far within the RMS area comes from Lake Holzmaar in the German Eifel region and the Belgian Scheldt basin. Within Lake Holzmaar the 10.3 ka event is reflected by increased allochtonous input pointing to increased soil (slope) erosion by water run-off [61]. According to Dreibrodt et al. [62] the latter is linked to repeated wildfires in the pine dominated forests, creating bare surfaces prone to erosion. These wildfires would have been triggered by the palaeoclimatic deterioration of the 10.3 ka event, a mechanism which came to an end with the establishment of mixed oak forests immediately following the climatic event. A similar observation has recently been reported at Kerkhove in the Scheldt basin, situated in the western part of the RMS-area [63]. Here, a slope deposit dated between ca. 11,200 and 9600 cal BP, was attested at the base of a levee, associated with high frequencies of pine pollen and microcharcoal. Although the link with the 10.3 ka event is less evident at Kerkhove, both sites are important as they demonstrate the occurrence of repeated wildfires during the (late) preboreal and early Boreal, when the landscape was dominated by coniferous forests, which is consistent with observations elsewhere in northern Europe [64]. These fires definitely must have impacted the human resources, but it is currently difficult to determine the precise extent.

A similar broad chronological coincidence is observed with respect to the sudden appearance of invasively retouched points (“Sonnisse Heide” and “Gelderhorsten” ATs) and trapezes (“Paardsdrank” and “Ruiterskuil” ATs), resp. with the 9.3 ka or IRD 6 event [65] and the 8.2 ka or IRD 5 event [66, 67] (Fig 3). The former microliths turned up between ca. 9390 and 9275 cal BP (68.2%), which is synchronic or slightly earlier than the start of the 9.3 ka event dated between ca. 9300 and 9190 cal BP [31]. By the end of this climatic event they had already fully replaced the older microlithic types, i.e. crescents and triangles. The shift from assemblages dominated by invasively retouched microliths to assemblages in which trapezes prevail clearly occurred during the 8.2 ka event dated between ca. 8250 and 8090 cal BP [31]; the former ended right before the start of this climatic oscillation (ca. 8440–8355 cal BP; 68.2%), while the latter started just after (ca. 7940–7875 cal BP) or maybe even during it (cf. Results). Contrary to the 10.3 ka event, both these younger climatic events, which lasted between 100 and 150 years and were accompanied by a drop of 1° to 2°C in mean temperature, are supposed to have led to increased drought. The diatom record and microstructure of the varves within the Lake Holzmaar [68] clearly point to drier winters and shorter and cooler summers, especially during the 8.2 ka event, which is consistent with observations elsewhere in northern Europe [62, 69]. Increased dryness is also expressed by reduced fluvial activity in most river valleys of the RMS area. There is little evidence of flood deposits at the time of these climatic oscillations [70, 71]. Furthermore, there is plenty of evidence of a change in the aggradation of palaeochannels; in many abandoned channels, in particular of small to medium sized rivers, peat accumulation considerably slowed down or even ceased during the (second half of the) Boreal-start of the Atlantic, pointing to a marked lowering of the water level [44, 72, 73]. Drier conditions are also reflected in the sporadic occurrence of macrofossil remains of marsh and riparian plants, the decrease in abundance of pollen and spores of riparian plants, and also in the drastic reduction of microfossils from fresh water algae and the scarcity of aquatic plants in both the micro- and macrofossil record. Whether all this results from the coldness and dryness of the climatic oscillations or is due to the increasing dominance of the high water consuming pine trees (or a combination of both) remains to be investigated. However, the latter seems highly unlikely for the southern loamy part of the RMS area (France, S Belgium) as pines were already outcompeted by hazel and oak, and to a lesser degree elm, by the middle of the Boreal [49, 74]. On the other hand, in the northern sandy lowland of the RMS (N Belgium, S Netherlands) pines may still have had an impact on ground water levels during the late Boreal and early Atlantic, as they persisted longer and often in a dominant position [75]. The longer survival of pines might also have prolonged the period of wildfires in these lowlands, as indirectly suggested by anthracological analyses of burnt ant-nests [76]. Though the exact amplitude and equifinality of the discussed environmental changes, such as the cooler and drier climatic conditions, decreasing water availability and increasing local wildfires, still need to be investigated in much more detail, it can be reasonably assumed that they had a considerable effect on the availability and distribution of subsistence resources of Mesolithic hunter-gatherers from the late Boreal and early Atlantic. Meanwhile, the sea level continued to rise spectacularly until at least 8500–8000 cal BP, the moment that the British island became separated from the continent (Fig 5). The latter is confirmed by the total lack of typical trapezes in Britain, a microlith type which, according to our chronological model, had reached the North Sea coast at the latest by ca. 8000/7900 cal BP and probably a few centuries earlier. By that time, another ca. 48.000 km^2^ of the North Sea basin was lost for human occupation [52], probably forcing people to migrate even further inland. In this context, it is interesting to mention the “Honey Hill” AT, present in central England [14, 54] (Fig 4). This still badly documented AT is characterized by the presence of leaf-shaped microliths with inverse basal retouch. Although they are comparable to the continental invasively modified microliths, in particular the sub-type with rounded base, they differ as the tip is still manufactured in the “old” fashion, i.e. by means of a direct, steep retouch. The continental specimens on the other hand are fully manufactured by means of the “new” technique of inverse and flat retouching. This technological difference may at first glance seem unimportant, yet it might indicate that the British “Honey Hill” AT represents an initial phase in the development of invasively retouched microliths. Unfortunately, the meagre radiocarbon evidence for the Honey Hill AT does not allow to verify this hypothesis, although recent excavations at Asfordby in Leicestershire yielded a coherent series of very old dates starting from ca. 10,200/9850 cal BP [77]. These dates push the origin of the invasively retouched points in Britain roughly a millennium further in time compared to the continent. However, as all dates are performed on calcined bones they need to be considered carefully knowing the problems inherent to this dating material [22, 23]. Furthermore, it needs to be taken into account that the lithic assemblage from Asfordby is potentially mixed, as it also includes numerous “older” types of microliths, such as obliquely truncated points and scalene triangles. Independent of this dating issue, the “Honey Hill” AT points to some kind of cross-channel connection during the final phase of inundation of the North Sea basin. Interestingly, this AT is spatially limited to the British Midlands and East Anglia [14], situated at the same height as the Doggerland uplands, representing the last land bridge with the continent (Fig 5). So it is not unlikely that the “Honey Hill” AT is linked to the last occupation of Doggerland, right before its final drowning.

Considering the evidence presented above, there is reason to believe that the invasively retouched microliths were conceived in response to these increasing environmental and demographic changes and served the same symbolic purpose as the microliths from the late 11th to mid 10th millennium. Recent micro-wear studies [34] have provided proof of their exclusive use as arrowheads, replacing older types, e.g. the unilateral points and points with retouched base (cf. supra). Interestingly these morphological changes were not limited to the arrow tip, but also involved the stone barbs. More or less synchronic with the appearance of the first invasively retouched microliths, the older types of barbs, e.g. crescents and triangles, were radically replaced by small backed bladelets resulting in a totally different design of the hunting equipment. Despite the absence of experimental research, it seems that this new hunting device did not provide any obvious functional advantages. On the contrary, the application of the invasive and bifacial retouch resulted in a considerable thinning of the arrow tips, making these new projectile types even more fragile on impact [34]. This observation reinforces the idea that the microlith forms, which appeared around the 9.3 ka event, were developed for social rather than functional reasons. There is currently little evidence to support the idea that these new microliths were a response to changing hunting strategies and/or game, as there is no marked difference in the faunal composition between Early and Middle Mesolithic sites. Both are characterized by an absolute predominance of wild boar (*Sus scrofa scrofa*), representing between 55% and 100% of the hunted assemblage [44]. The focus on wild boar hunting is generally explained by the increased availability of the species, due to their very high reproduction rate and forest expansion [78]. At most Early and Middle Mesolithic sites red deer (*Cervus elaphus*) is the second best represented species amounting to between 10% and 20%. On some sites, fur-bearing species, mainly beaver (*Castor fiber*), are also important (ca. 20%). The only possible subsistence change during the Middle Mesolithic might be the gradually increasing importance of freshwater fishing, although the current data do not allow to assess the contribution of fish to the diet precisely [34, 44].

Furthermore, the theory of symbolic tools is strengthened by the spatial distribution of invasively retouched microliths, which is much more confined compared to the older ATs (Fig 4). Their spread is limited to the area between the Seine in the south and the Rhine/Meuse in the north and east, covering ca. 150,000km^2^ [33, 79]. It thus looks as if the 9.3 ka event together with the advanced North Sea inundation intensified the need for a passive and symbolic protection of the social territory boundaries. This makes the RMS area unique, as no clear changes in projectile design and technology have been reported elsewhere in Europe around the timing of the 9.3 ka event [54, 80, 81].

Contrary to the invasively retouched microliths which were invented and exclusively used within the RMS area, the trapeze-shaped microliths, which indicate the start of the Late Mesolithic “Paardsdrank” and “Ruiterskuil” ATs, were clearly introduced from other regions in Europe or even further away. Furthermore their appearance is synchronic with a major technological change characterized by the production of very regular blade(let)s, named Montbani-blades, by means of indirect percussion and/or the pressure technique. Possible centers of origin of this blade-and-trapeze technology are situated in NW Africa [82, 83], SE Europe [84] and/or maybe even further in eastern Asia [85]. Although the mechanisms of diffusion of this new technology remain to be investigated (demic diffusion, technological transmission, …), it is obvious that these new projectile implements testify of new hunting traditions and that they offered several functional advantages thanks to their versatility compared to previous microliths. They could be mounted as arrowheads, either transversally or axially, but could also be used as barbs. Furthermore, mounted in a transversal way they provided the arrows with a much larger and sharper cutting edge which was more resistant to impact and created larger wounds [6, 86]. The scarcity of Late Mesolithic faunal remains within the RMS area hinders to investigate whether there is a link with changes in hunted game species. The few sites dated to the late 9^th^ and 8^th^ millennium cal BP which yielded faunal remains [87, 88, 89] suggest that wild boar often remained the prime prey species, although on some sites red deer was almost as important or even dominant. However, it is questionable whether the hunting of red deer was the trigger for the introduction and use of trapezes within the RMS area. Either way, it cannot explain the initial development of this new type of armature and knapping technology in distant areas (cf. supra) with totally different climates and environments. Yet, in the Maghreb (NW Africa), the appearance of the blade-and-trapeze technology seems also closely related to a major shift in hunting practices, from a focus on large (*Bos*, *Equus*) to smaller game (*Gazella*). Interestingly, this abrupt subsistence shift is linked to major environmental changes, i.e. increased aridity, resulting from the 8.2 ka event [82].

The appearance of the first trapezes along the southern North Sea basin also roughly coincides with a series of punctuated environmental events which definitely must have altered the lives of hunter-gatherers. First there was the mega-tsunami, generated by the Second Storegga Slide along the west coast of Norway around ca. 8100 cal BP [90] and, the rapid vertical jump of the sea-level of 2.11 ± 89 m along the Dutch coast between ca. 8.45 and 8.25 cal BP as a result of the two-staged drainage of the Laurentide proglacial Lakes Agassiz and Ojibway [1, 91]. However, the effects of both these events will have been limited to the coastal and delta areas. In addition, the drowning of the North Sea basin after 8000 cal BP considerably slowed down; between 8000 and 7000 cal BP ca. 15,000 km^2^ was lost, which is substantially less than before, impacting the distribution of hunter-gatherers to a lesser degree. Probably the cooler and drier conditions resulting from the 8.2 ka event had a more substantial impact on the latter. Environmental studies in southern Scandinavia [92, 93] and western Ireland [94] have pointed to a reduction of temperate thermophilous tree taxa, such as hazel (*Corylus*), oak (*Quercus*), alder (*Alnus*) and elm (*Ulmus*), in favor of cool-tolerant taxa, such as birch (*Betula*) and pine (*Pinus*), in response to this climatic anomaly. Knowing that hazelnuts and other fruit- and nut-bearing shrubs were intensively exploited by Mesolithic hunter-gatherers [51] their availability might have been considerably reduced. Although similar vegetational signals have not (yet) been found in the pollen records of the RMS region, anthracological analysis of burnt ant-nests provides indirect evidence of a temporal reappearance of pine within mixed deciduous forests, starting from roughly 8200 cal BP. It also suggests that this temporal return of pines re-activated wildfires on a local level [76]. If the introduction of the blade-and-trapeze technology within the RMS area was really a response to the environmental changes triggered by the 8.2 ka cooling, this implies that these new hunting implements might also have served the maintenance of social boundaries. Although the general design of the trapezes found all over Europe is very similar, some subtle regional differences are noticed which might hint at their use in the context of social boundary defense. Earlier studies [33, 95, 96] have pointed out the different lateralization, i.e. position of retouched edge, of trapezes found along both sides of the Seine River (Fig 4). The trapezes south of the Seine are almost exclusively retouched along their left side while the northern ones, belonging to the RMS area, in general have a right lateralization. It is thus possible that from the end of the 9th millennium cal BP onwards armature lateralization instead of general morphology played an important role in the context of maintaining the existing social boundaries in response to environmental changes.

Besides resource stress caused by the 8.2 ka event, other more long-term factors may have contributed to increased territoriality and social boundary defense during the Late Mesolithic. Within several regions of the RMS area a marked decline in sites has been reported, which is tentatively interpreted as reflecting decreasing residential mobility possibly resulting from a change from dispersed to more clustered subsistence resources and/or an increased importance of fishing [56]. While the coniferous forests of the (Pre)boreal were still rather open allowing the growth of dense undergrowth vegetation, during the Atlantic they evolved into dense and dark deciduous forests characterized by a concentration of subsistence resources, including fruit- and nut-bearing trees and shrubs and wild game, along forest edges and small forest openings, the former generally coinciding with the dry banks of the river floodplains. From ethnography [45–47] it is known that territoriality often develops in regions with predictable dense and clustered resources, as these are crucial to the survival of the group and are thus worth defending against a relatively low cost. Anyhow, it is clear that within the RMS area, as well as in other European regions, the shift to trapezes as main hunting projectiles was most likely due to a combination of different regional factors.

## Conclusions

In this paper it is argued that the development of radically new hunting projectiles in the course of the Mesolithic along the southern North Sea basin had probably more to do with social boundary defense than with functional optimalization of the hunting equipment. Both long-term and short-term climatic and environmental changes may have resulted in resource stress and competition, which triggered increased territoriality among contemporaneous hunter-gatherers. The continuous and steep rise in sea levels as a result of Early Holocene warming most likely induced movements of whole population groups from an area greater than 100,000km^2^, making the RMS area one of the most impacted regions by sea level rises within Early Holocene Europe. These forced migrations presumably led to increased population densities and subsequent resource stress and competition in the territories adjacent to the drowning North Sea basin. In addition, competition may have been reinforced by the occurrence of a series of punctuated cooling events. Statistical modelling of critically selected radiocarbon dates has demonstrated a broad chronological correspondence between at least three radical changes in the design and technology of stone arrowheads and barbs and short but abrupt cooling events. Although strict synchronicity cannot (yet) be proven due to chronological imprecision of both the climatic and archaeological records, the fact that changes in microlith design in all three cases situate close to the timing of these cooling events, increases the potential of possible causality. Similarly, the lack of high-resolution, centennial-to-decadal environmental data currently hinders a full assessment of the impact of these short climatic events; however there are several indications that cooling resulted in increased wetness (10.3 ka event) or drought (9.3 and 8.2 ka events) and wildfires, which most likely impacted the availability and distribution of different subsistence resources. It thus seems that the Pleistocene to Holocene transition was much more dynamic in terms of environmental and human changes than previously thought. Mesolithic research in the RMS area now needs a more holistic approach in order to determine whether these climatic and environmental changes also affected other aspects of Mesolithic behavior, such as the knapping technology, raw material procurement and distribution, tool function, settlement system and land-use.

## Supporting information

S1 Table List of the selected radiocarbon dates. (DOC)

S2 Table Bayesian model 1. (XLSX)

S3 Table Bayesian model 2. (XLSX)

S4 Table Bayesian model 3. (XLSX)
