# Supplementary material for: Mesolithic projectile variability along the southern North Sea basin (NW Europe): Hunter-gatherer responses to repeated climate change at the beginning of the Holocene
Source: PLoS One. 2019 Jul 17;14(7):e0219094. doi: 10.1371/journal.pone.0219094 (PMC6636730; doi:10.1371/journal.pone.0219094)
Supplement: S1 Table — (DOC) [file pone.0219094.s001.doc]

| Archaeological Site | Laboratory Code | ^14^C date BP | Sample Composition | Comments | Reference |
| --- | --- | --- | --- | --- | --- |
| **NEERHAREN ASSEMBLAGE TYPE** | | | | | |
| Verrebroek-Dok 1-9 | UtC-7851 | 9130±75 | Charred hazelnut shell |  | *97* |
| Verrebroek-Dok 1-14(70) | UtC-9223 | 9080±60 | Charred hazelnut shell |  |  |
| Verrebroek-Dok 1-14(70) | UtC-9418 | 9060±70 | Charred hazelnut shell |  |  |
| Verrebroek-Dok 1-14(70) | UtC-9225 | 9270±60 | Charred hazelnut shell |  |  |
| Verrebroek-Dok 1-17 | UtC-7120 | 9270±50 | Charred hazelnut shell |  |  |
| Verrebroek-Dok 1-17 | UtC-7119 | 9280±50 | Charred hazelnut shell |  |  |
| Verrebroek-Dok 1-17 | UtC-7118 | 8930±60 | Charred hazelnut shell |  |  |
| Verrebroek-Dok 1-22 | UtC-8389 | 9310±40 | Charred hazelnut shell |  |  |
| Verrebroek-Dok 1-22 | UtC-8393 | 9210±40 | Charred hazelnut shell |  |  |
| Verrebroek-Dok 1-22 | UtC-9438 | 9290±80 | Charred hazelnut shell |  |  |
| Oostwinkel- Mostmolen | UtC-3438 | 9250±160 | Charred hazelnut shell |  | *98* |
| Neerharen- De Kip | Lv-1092 | 9170±100 | Charred hazelnut shell |  | *99* |
| Posterholt | UtC-4915 | 9160±80 | Charred hazelnut shell |  | *100* |
| Posterholt | UtC-4916 | 9100±50 | Charred hazelnut shell |  |  |
| Posterholt | UtC-4917 | 9080±50 | Charred hazelnut shell |  |  |
| Posterholt | UtC-4914 | 8800±60 | Charred hazelnut shell | Outlier (AI=50.4%) |  |
| Rueil IV | GrA-13513 | 9080±50 | Animal bone |  | *101* |
| Rueil IV | GrA-13404 | 9510±50 | Animal bone |  |  |
| Rueil IV | GrA-13974 | 9430±50 | Animal bone |  |  |
| Reuil IV | GrA-12129 | 8130±130 | Animal bone | Outlier (AI=3.8%) |  |
| Warluis Va1 | Erl-10712 | 9390±69 | Bone (*Sus scrofa*) |  | *102* |
| Warluis Va2 | Erl-10713 | 9278±68 | Bone (*Cervus elaphus*) |  |  |
| Rotterdam- Maasvlakte 2 | GrA-56453 | 9215±45 | Indetermined bone |  | *103* |
| Rotterdam- Maasvlakte 2 | GrA-56454 | 9205±45 | Bone (*Sus scrofa*) |  |  |
| Rotterdam- Maasvlakte 2 | GrA-55485 | 8920±45 | Charred hazelnut shell |  |  |
| Greenham Dairy Farm | OxA-5194 | 9120±80 | Charred hazelnut shell |  | *14* |
| Lackford Heath | OxA-2342 | 9240±110 | Resin block |  |  |
| Marsh Benham | OxA-5195 | 8905±80 | Charred hazelnut shell |  |  |
| Oakhanger VII | Q-1489 | 9225±170 | Charred hazelnut shell |  |  |
| Thatcham III | OxA-2848 | 9200±90 | Resin on flake |  |  |
| Eton Rowing Lake | OxA-14088 | 9540±45 | Bone (*Bos Primigenius*) |  | *54* |
| Three Ways Wharf | OxA-5557 | 9280±110 | Tooth (*Cervus elaphus*) |  |  |
| Three Ways Wharf | OxA-5558 | 9265±80 | Tooth (*Capreolus capreolus*) |  |  |
| Three Ways Wharf | OxA-5559 | 9200±75 | Tooth (*Cervus elaphus*) |  |  |
| ***OURLAINE ASSEMBLAGE TYPE*** | | | | | |
| Verrebroek-Dok 1-1 | UtC-3915 | 9110±65 | Charred hazelnut shell |  | *97* |
| Verrebroek-Dok 1-2.1 | UtC-8398 | 9265±40 | Charred hazelnut shell |  |  |
| Verrebroek-Dok 1-4 | UtC-8397 | 9065±40 | Charred hazelnut shell |  |  |
| Verrebroek-Dok 1-14 | UtC-7046 | 9100±60 | Charred hazelnut shell |  |  |
| Verrebroek-Dok 1-14 | UtC-7045 | 9230±50 | Charred hazelnut shell |  |  |
| Verrebroek-Dok 1-18 | UtC-9224 | 9160±60 | Charred hazelnut shell |  |  |
| Verrebroek-Dok 1-22(67) | UtC-8388 | 8755±40 | Charred hazelnut shell |  |  |
| Verrebroek-Dok 1-23 | UtC-9228 | 9020±60 | Charred hazelnut shell |  |  |
| Verrebroek-Dok 1-39 | NZA-11015 | 8900±90 | Charred hazelnut shell |  |  |
| Verrebroek-Dok 1-44(91) | NZA-11248 | 8755±85 | Charred hazelnut shell |  |  |
| Verrebroek-Dok 1-49 | NZA-11249 | 8675±55 | Charred hazelnut shell |  |  |
| Verrebroek- Aven Ackers | KIA-38505 | 9090±45 | Charred hazelnut shell |  | *104* |
| Verrebroek- Aven Ackers | KIA-38499 | 9005±40 | Charred hazelnut shell |  |  |
| Verrebroek- Aven Ackers | KIA-38506 | 8805±40 | Charred hazelnut shell |  |  |
| Doel- Deurganckdok J/L-C2 | KIA-20470 | 8830±45 | Charred hazelnut shell |  | *105* |
| Doel- Deurganckdok J/L-C3 | KIA-24034 | 8630±60 | Charred hazelnut shell |  | *106* |
| Doel- Deurganckdok J/L-C3 | KIA-24454 | 8485±40 | Charred hazelnut shell |  |  |
| Doel- Deurganckdok J/L-C3 | KIA-43577 | 8525±40 | Charred hazelnut shell |  |  |
| Doel- Deurganckdok J/L-C3 | KIA-30962 | 8965±45 | Charred hazelnut shell | Statistical outlier |  |
| Warluis IVd | Erl-10722 | 9111±70 | Charred hazelnut shell |  | *102* |
| Saleux 244b | GrA-13976 | 9190±50 | Diaphyse indetermined |  | *42* |
| Hangest-Gravière IIN | Gif-9328 | 9100±80 | Bones red deer, wild boar, roe deer | Possibly contaminated with older bone | 42 |
| Hangest-Gravière IIN | Gif-8912 | 8830±90 | Charred hazelnut shell |  |  |
| Saleux-Les Baquets 125 | GrA-21032 | 8930±50 | Human bone |  | *42* |
| Saleux-Les Baquets 125 | OxA-7615 | 8695±65 | Bone (*Sus scrofa*) |  |  |
| Warluis IIc | GrA-23542 | 9090±70 | Bone (*Sus scrofa*) |  | *102* |
| Warluis IIc | GrA-27847 | 9000±50 | Charred hazelnut shell |  |  |
| Warluis IIc | GrA-23549 | 8760±60 | Bone (*Sus scrofa*) |  |  |
| Warluis IIc | GrA-23550 | 8830±60 | Bone (*Sus scrofa*) |  |  |
| Warluis IId | GrA-23537 | 8510±70 | Bone (*Sus scrofa*) |  |  |
| Saleux-Les Baquets 244a | GrA-18829 | 8700±60 | Bone (*Sus scrofa*) |  | *42* |
| Saleux- Les Baquets 244a | GrA-13407 | 8670±50 | Bone (*Sus scrofa*) |  |  |
| Saleux-Vierge Catherine - Niv. Inf. | OxA-4929 | 8645±70 | Bone (*Sus scrofa*) |  |  |
| Rosnay- Haute-de-Vallière | GrA-51793 | 9280±40 | Charred hazelnut shell |  | *107* |
| Rotterdam- Maasvlakte 2- Trench 1 | GrA-55482 | 7820±45 | Charred hazelnut shell | Outlier (AI=7.7%) | 103 |
| ***VERREBROEK ASSEMBLAGE TYPE*** | | | | | |
| Verrebroek-Dok 1-2.2 | UtC-8391 | 8850±50 | Charred hazelnut shell |  | *97* |
| Verrebroek-Dok 1-6 | UtC-8961 | 9165±45 | Charred hazelnut shell |  |  |
| Verrebroek-Dok 1-6 (S16) | UtC-3439 | 9150±100 | Charred hazelnut shell |  |  |
| Verrebroek-Dok 1-7 (S14) | UtC-3451 | 9120±120 | Charred hazelnut shell |  |  |
| Verrebroek-Dok 1-7 | UtC-8395 | 9015±40 | Charred hazelnut shell |  |  |
| Verrebroek-Dok 1-14 (71) | UtC-9419 | 9070±70 | Charred hazelnut shell |  |  |
| Verrebroek-Dok 1-41 | NZA-11012 | 9180±60 | Charred hazelnut shell |  |  |
| Haelen-Broekweg | KIA-17631 | 9020±50 | Charred hazelnut shell |  | *108* |
| Haelen-Broekweg | KIA-17612 | 9060±45 | Charred hazelnut shell |  |  |
| Bazel-Stuw- C2 | RICH-26075 | 8587±39 | Charred hazelnut shell |  | *unpublished* |
| Bazel-Stuw- C2 | RICH-26076 | 8782±38 | Charred hazelnut shell |  |  |
| Bazel-Stuw- C2 | RICH-26077 | 8646±44 | Charred hazelnut shell |  |  |
| Bazel-Stuw- C2 | RICH-26078 | 8679±37 | Charred hazelnut shell |  |  |
| ***CHINRU ASSEMBLAGE TYPE*** | | | | | |
| Verrebroek-Dok 1-14 (72) | UtC-7252 | 8750±40 | Charred hazelnut shell |  | *97* |
| Verrebroek-Dok 1-14 (68) | UtC-9222 | 8400±60 | Charred hazelnut shell |  |  |
| Verrebroek-Dok 1-16 | UtC-7117 | 8850±40 | Charred hazelnut shell |  |  |
| Verrebroek-Dok 1-28 (99) | UtC-9226 | 8810±60 | Charred hazelnut shell |  |  |
| Verrebroek-Dok 1-28 | UtC-9433 | 8800±80 | Charred hazelnut shell |  |  |
| Verrebroek-Dok 1-44 (92) | NZA-11017 | 8790±60 | Charred hazelnut shell |  |  |
| Saleux- Les Baquets 295 | Beta-170947 | 8590±40 | Bone (*Sus scrofa*) |  | *42* |
| Saleux- Les Baquets 295 | Beta-191693 | 8510±50 | Bone (*Sus scrofa*) |  |  |
| Saleux- Les Baquets 295 | Beta-170948 | 8310±40 | Bone (*Sus scrofa*) |  |  |
| Saleux- Les Baquets 295 | Beta-191694 | 8210±50 | Bone (*Castor fiber*) |  |  |
| Kerkhove-Stuw-1b | RICH-23847 | 8859±35 | Charred hazelnut shell |  | *34* |
| Kerkhove-Stuw-6 | RICH-23841 | 8803±38 | Charred hazelnut shell |  |  |
| Kerkhove-Stuw-6 | RICH-23838 | 8796±40 | Charred hazelnut shell |  |  |
| Kerkhove-Stuw-11 | RICH-23839 | 8860±37 | Charred hazelnut shell |  |  |
| ***SONNISSE HEIDE/GELDERHORSTEN ASSEMBLAGE TYPE*** | | | | | |
| Belloy- Plaisance | Gif-8705 | 8240±100 | Undetermined bone |  | *42* |
| Hangest III-2/3a | Gif-9276 | 8290±70 | Charred hazelnut shell |  | *42* |
| Saleux-Vierge Catherine- Niv. Sup. | OxA-6203 | 8210±110 | Bone (*Bos primigenius*) |  | *42* |
| Chaussée-Tirancourt-pit2 | Gif-8913 | 7840±90 | Charcoal |  | *102* |
| Verrebroek- Aven Ackers | KIA-38503 | 7865±35 | Charred hazelnut shell |  | *104* |
| Verrebroek- Aven Ackers | KIA-38497 | 7770±40 | Charred hazelnut shell |  |  |
| Verrebroek- Aven Ackers | KIA-38504 | 7755±35 | Charred hazelnut shell |  |  |
| Verrebroek- Aven Ackers | KIA-38498 | 7710±35 | Charred hazelnut shell |  |  |
| Verrebroek- Aven Ackers | KIA-38500 | 7660±35 | Charred hazelnut shell |  |  |
| Verrebroek- Aven Ackers | KIA-31351 | 8070±55 | Charred hazelnut shell |  |  |
| Remilly-les-Pothées-9 | Beta-383251 | 8200±30 | Charred hazelnut shell |  | *109* |
| Remilly-les-Pothées-9 | Beta-401970 | 8180±30 | Charred hazelnut shell |  |  |
| Remilly-les-Pothées-8 | Beta-383255 | 7950±40 | Charred hazelnut shell |  |  |
| Liege-Place St-Lambert secteur DDD- unité VIIB | OxA-8943 | 7745±60 | Calcaneus (*Cervus elaphus*) |  | *110* |
| Liege-Place St-Lambert secteur DDD | OxA-4781 | 7850 75 | Metatarsal (*Cervus elaphus*) |  |  |
| ***PAARDSDRANK/RUITERSKUIL ASSEMBLAGE TYPE*** | | | | | |
| Remilly-les-Pothées-2 | Beta-383262 | 6650±30 | Charred hazelnut shell |  | *109* |
| Remilly-les-Pothées-2 | Beta-401972 | 6580±30 | Charred hazelnut shell |  |  |
| Remilly-les-Pothées-2 | Beta-401971 | 6550±30 | Charred hazelnut shell |  |  |
| Remilly-les-Pothées- St. 11416-6 | Beta-383260 | 6560±30 | Charred hazelnut shell |  |  |
| Remilly-les-Pothées- St. 11416-16 | Beta-383257 | 6550±30 | Charcoal indetermined |  |  |
| Remilly-les-Pothées- St. 11416-12 | Beta-383256 | 6410±30 | Charcoal Corylus |  |  |
| Remilly-les-Pothées- St. 11529-493 | Beta-383258 | 6580±30 | Charred hazelnut shell |  |  |
| Remilly-les-Pothées- St. 11529-491 | Beta-383259 | 6520±30 | Charred hazelnut shell |  |  |
| Godinne-Abri de Chauveau- layer MG-MR | LV-1615 | 7350 ±75 | Human bones | Outlier (AI=46.3%) | *111* |
| Remouchamps- Station Leduc | LV-1401 | 6990± 90 | Charred hazelnut shell |  | *112* |
| Weelde-Paardsdrank 5 | LV-959 | 6990± 135 | Charred hazelnut shell |  | *113* |
| Modave-Trou Al’Wesse- layer 4b-δ | LV-1751 | 6650± 70 | Undetermined bones |  | *114* |
| Modave-Trou Al’Wesse- layer 4b-δ | Beta-251056 | 6890± 40 | Bone (*Bos primigenius*) with  butchery traces |  |  |
| Concevreux | Beta-282245 | 7070±40 | Tooth (*Sus scrofa*) |  | *115* |
| Verrebroek-Aven Ackers | KIA-37 694 | 6785±40 | Charred hazelnut shell |  | *116* |

Table 1: List of the selected radiocarbon dates.

**References**

97. Crombé Ph. (ed.) *The last hunter-gatherer-fishermen in Sandy Flanders (NW Belgium); the Verrebroek and Doel excavation projects, Part 1: palaeo-environment, chronology and features.* Archaeological Reports Ghent University, Gent; 2005.

98. Crombé Ph. *The Mesolithic in Northwestern Belgium, Recent Excavations and Surveys*. Archaeopress, Oxford; 1998.

99. Lauwers R, Vermeersch PM. Un site du Mésolithique ancient à Neerharen-De Kip. *Contributions to the study of the Mesolithic of the Belgian Lowland*, Vermeersch PM editor. Studia Praehistorica Belgica, Tervuren; 1982. p. 15-52.

100. Verhart LBM. An Early Mesolithic hunting camp at Posterholt, municipality of Ambt Montfort (the Netherlands). *Mesolithic Miscellany* 1995; 16: 20-29.

101. Lang L, Sicard S. Les occupations mésolithiques des Closeaux à Rueil-Malmaison (Hauts-de-Seine). *Mém. Soc. Préh. Fran.* 2008 ; XLV: 63-83.

102. Ducrocq T. Le Beuronien à segments dans le Nord de la France. Prémices d’une approche palethnologique. *Séances Soc. Préh. Fran.* 2013 ; 2: 189-206.

103. Moree JM, Sier MM. (eds.) *Interdisciplinary Archaeological ,Research Programme Maasvlakte 2, Rotterdam*. BOOR, Rotterdam ; 2015.

104. Crombé Ph, Sergant J, Lombaert L, Van Strydonck M, Boudin M. The Mesolithic and Neolithic site of Verrebroek - Aven Ackers (East Flanders, Belgium): the radiocarbon evidence. *Notae Praehistoricae* 2009 ; 29: 15-21.

105. Jacops J, Noens G, Crombé Ph. Onderzoek van een vroegmesolithsche concentratie te Doel-Deurganckdok (zone J/L, concentratie 2). *Notae Praehistoricae* 2007; 27: 75-81.

106. Noens G. Analyse intrasite de gisements du Mésolithique ancien de la Flandre sableuse : l’exemple de Doel « Deurganckdok J/L », C3. *Séances Soc. Préh. Fran*. 2013 ; 2: 217-234.

107. Souffi B, Guéret C, Griselin S, Guillemard I, Leduc C. Le site Mésolithique de Rosnay « Haut-de-Vallière » (Marne) : une occupation spécialisée du premier Mésolithique. *Bull. Soc. Préh. Fran.* 2015 ; 112: 717-759.

108. Bats M, et al. Onderzoek langs de omleiding N273 te Haelen (provincie Limburg, Nederland): Vroeg-Mesolithicum en Vroeg-Neolithicum. *Notae Praehistoricae* 2002; 22: 87-102.

109. Souffi B., Guéret C, Leduc C. Nouvelles données chronoculturelles et palethnographiques sur le Mésolithique des VIIIe et VIe millénaires dans le Nord de la France. Le site de « la Culotte » à Remilly-les-Pothées (Ardennes, France). *Bull. Soc. Préh. Fran.* 2018 ; 115: 531-565.

110. van der Sloot P, et al. Le Mésolithique et le Néolithique du site Saint-Lambert à Liège dans leur contextes chronologique, géologique et environnemental. Synthèse des données et acquis récents. *Notae Praehistoricae* 2003 ; 23: 79-104.

111. Toussaint M, Becker A. Recherches 1986 à l’abri sous roche de Chaveau, à Godinne, Yvoir. *Activités 86 à 87 du S.O.S. Fouilles* 1988 ; **5:** 65-72.

112. Gob A, Jacques M-C. A Late Mesolithic dwelling structure at Remouchamps, Belgium. *Journal of Field Archaeology* 1985 ; 12: 163-175.

113. Huyge D, Vermeersch PM. Late Mesolithic settlement at Weelde-Paardsdrank. *Contributions to the study of the Mesolithic of the Belgian Lowland*, Vermeersch PM editor. Studia Praehistorica Belgica, Tervuren; 1982. p. 115–209.

114. Miller R, Zwyns N, Otte M, Stevens C, Stewart J. La séquence mésolithique et néolithique du Trou Al’Wesse (Belgique) : résultats pluridisciplinaires. *L’Anthropologie* 2012 ; 116: 99-126.

115. Robert B, et al. Tombe à incinération du Mésolithique à Concevreux (Aisne). Revue Archéologique de Picardie 2015 ; 3-4: 15-32.

116. Robinson R, Lombaert L, Sergant J, Crombé Ph. Armatures and the question of forager-farmer contact along the North-western fringe of the LBK. The site of Verrebroek “Aven Ackers” (East Flanders, Belgium). *Archäologisches Korrespondenzblatt* 2011; 41: 473- 490.
